# Renal Leiomyoma in a Female Adult: A Histopathological Case Report

**DOI:** 10.7759/cureus.70898

**Published:** 2024-10-05

**Authors:** Anastasia I Bekyarova, Andreya Kirilova, Kristina Naydenova, Hristo Popov, George S Stoyanov

**Affiliations:** 1 General and Clinical Pathology, Forensic Medicine and Deontology, Medical University of Varna, Varna, BGR; 2 Pathology, Multiprofile Hospital for Active Treatment, Shumen, BGR

**Keywords:** benign mesenchymal tumor, kidney tumor, renal leiomyoma, renal tumor, spindle cell renal neoplasm

## Abstract

Renal leiomyomas are rare benign mesenchymal tumors that arise from the smooth muscle cells in the renal capsule, renal pelvis, and the smooth muscles of vessels in the kidney. They are usually found by accident during autopsies or on different imaging modalities made on other occasions. The clinical presentation may include hematuria and abdominal or flank pain, although renal leiomyomas are most frequently asymptomatic. This case concerns a 45-year-old woman with an asymptomatic tumor mass in the left kidney, discovered incidentally on an outpatient CT scan conducted on an unrelated occasion. Histological examination revealed well-demarcated tumor formation on regular hematoxylin and eosin (H&E) staining consisting of intersecting fascicles of spindle cells with blunt-ended, cigar-shaped nuclei and eosinophilic cytoplasm. The neoplasm showed no mitotic activity. Immunohistochemistry (IHC) was performed to exclude different spindle cell pathologies, such as solitary fibrous tumor of the kidney, primary leiomyosarcoma, and, less probably, sarcomatoid variants of renal cell carcinoma (RCC). Tumor cell cytoplasm was positive for h-caldesmon, smooth muscle actin (SMA), and desmin. The proliferation index Kiel-67 (Ki-67) was evaluated as less than 1%. Considering the microscopic description and the IHC results, the neoplasm was interpreted as a renal leiomyoma. In light of the rarity of these tumors, pathologists should consider differential diagnosis with precision. In challenging cases, immunohistochemical examination can be beneficial to distinguish between several spindle cell renal neoplasms, such as solitary fibrous tumor, leiomyosarcoma, sarcomatoid variant of RCC, fat-poor angiomyolipoma, benign peripheral nerve sheath tumors, renomedullary interstitial cell tumor, benign fibrous histiocytoma, primary angiosarcoma, mucinous tubular and spindle cell renal cell carcinoma (MTSRCC).

## Introduction

Leiomyomas of the kidney are rare benign mesenchymal tumors, affecting predominantly females (2:1) with an average age of 47 [1]. They arise from the smooth muscle cells in the renal capsule, renal pelvis, and the vascular smooth muscle of vessels in the kidney. Based on their exact location, these tumors can be subclassified into subcapsular, capsular, and subpelvic types [1]. Renal leiomyomas account for less than 0.5% of surgically treated renal neoplasms. Their incidence seems to be greater in autopsies (4-5.5%), where they are usually found by accident. Imaging modalities, mostly done on other occasions, allow the detection of asymptomatic formations. Some patients may experience unspecific manifestations such as hematuria and abdominal or flank pain [2,3]. Renal leiomyomas have broad differential diagnosis regarding spindle cell renal neoplasms that should be considered during histological examination. 

## Case presentation

This case study concerns a 45-year-old woman who was found to have an asymptomatic tumor mass in the left kidney. The formation was discovered incidentally during a CT scan conducted on an outpatient basis for reasons unrelated to this finding. An ambulatory sonogram showed left-sided peripheral heterogeneous, mainly iso- and hypoechogenic formation in the lower renal pole, which was 25 x 20 mm in size. The patient was referred for surgical treatment, and a robot-assisted partial resection of the affected kidney was performed. 

Grossly, the excised specimen represented a round-to-oval, well-circumscribed tumor mass with grayish-white coloration, measuring 25 mm at its greatest dimension, located in the parenchymal periphery. No invasion of the capsule and the perirenal tissue was observed.

**Figure 1:**
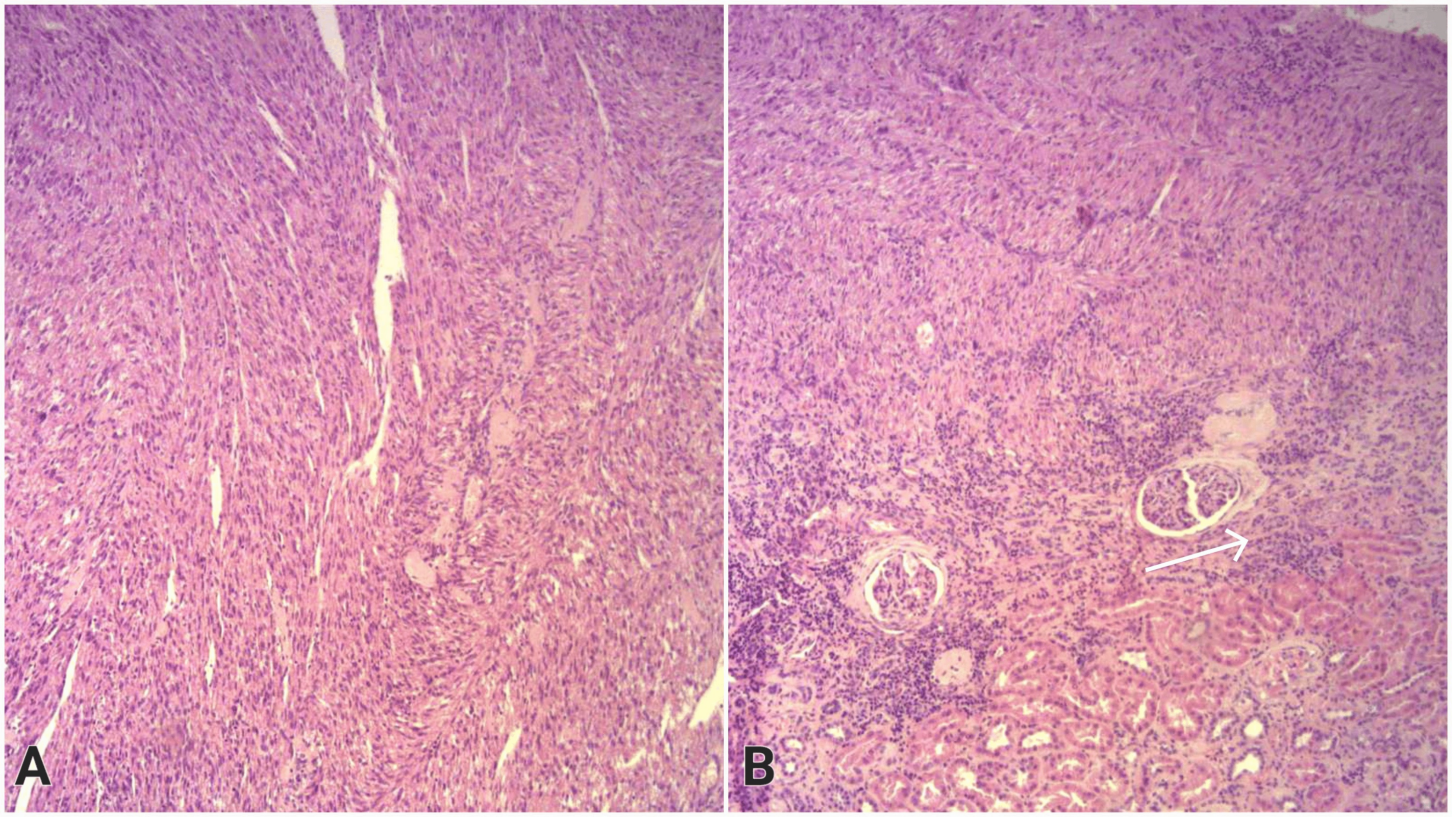
Histopathology of the renal leiomyoma, H&E stain, 100x A: Central view of the tumor showing intersecting bundles of spindle cells with cigar-shaped nuclei and eosinophilic cytoplasm; B: In the periphery, the renal tubules exert signs of atrophy (arrow) due to compression by the tumor mass H&E: Hematoxylin and eosin

Histological examination revealed well-demarcated tumor formation on regular hematoxylin and eosin (H&E) staining consisting of intersecting fascicles of spindle cells with blunt-ended, cigar-shaped nuclei and eosinophilic cytoplasm (Figure 1A). No mitoses were seen regardless of Kiel-67 (Ki-67) (proliferation index) being less than 1% positive. The surrounding renal tissue demonstrated atrophic changes (Figure 1B).

**Figure 2:**
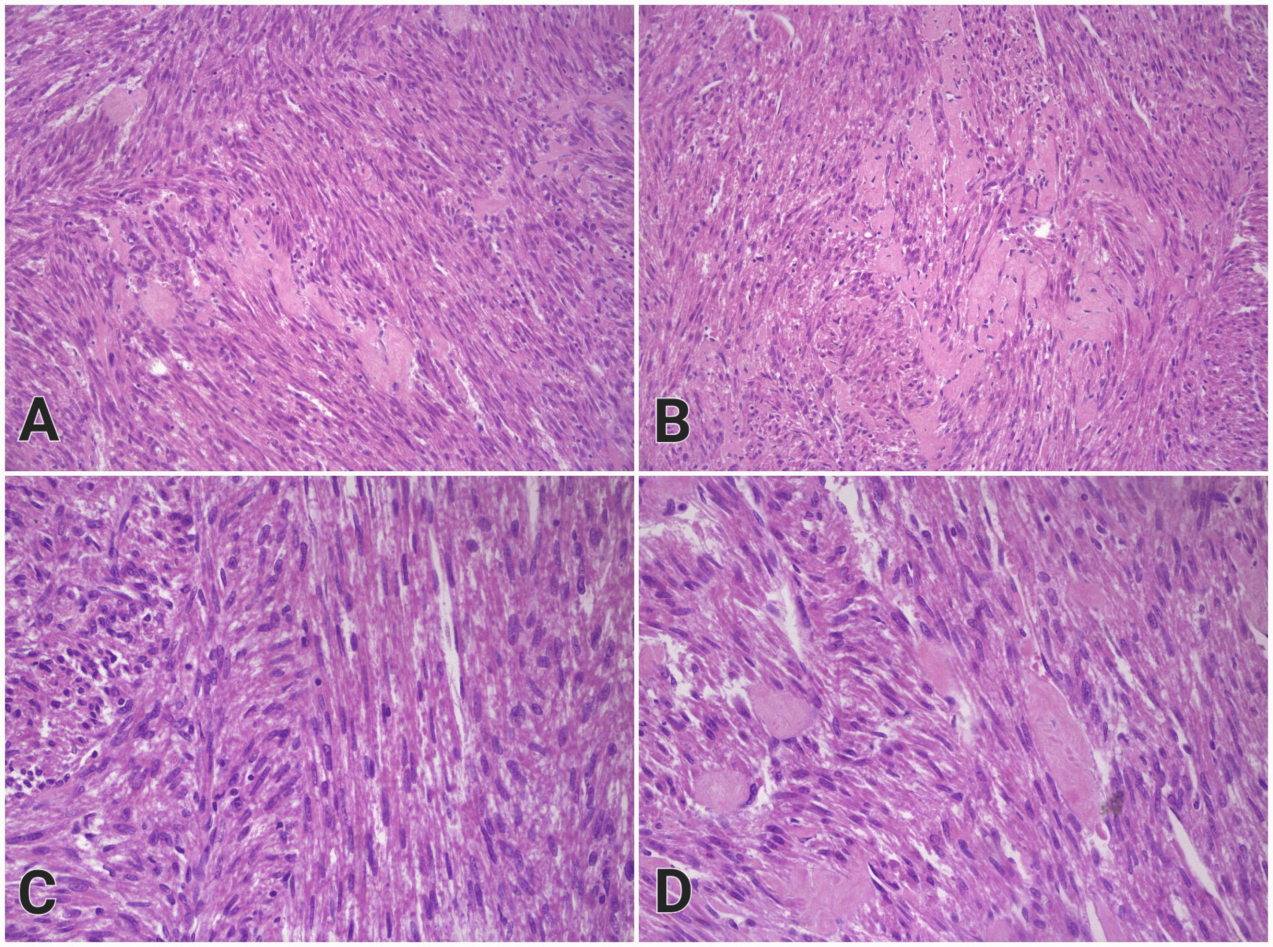
High magnification photos of the renal leiomyoma, demonstrating absence of cellular or nuclear atypia, H&E stain A: 200x magnification, B: 200x magnification, C: 400x magnification, D: 400x magnification In Figure 2A, the section crosses the tumor cell bundles longitudinally, and in Figure 2B, it is made transversely, resulting in a false loss of the spindle-shaped appearance of the cells. In Figure 2C, intersecting fascicles of tumor cells are shown, and in Figure 2D, in addition to the described histological picture, a homogeneous eosinophilic hyalinized substance is seen in the stroma. H&E: Hematoxylin and eosin

At higher magnification, the tumor cells did not show any cellular or nuclear atypia, which contributed to the benign appearance of the tumor (Figure 2). Moreover, no necrotic foci were found within the specimen.

**Figure 3:**
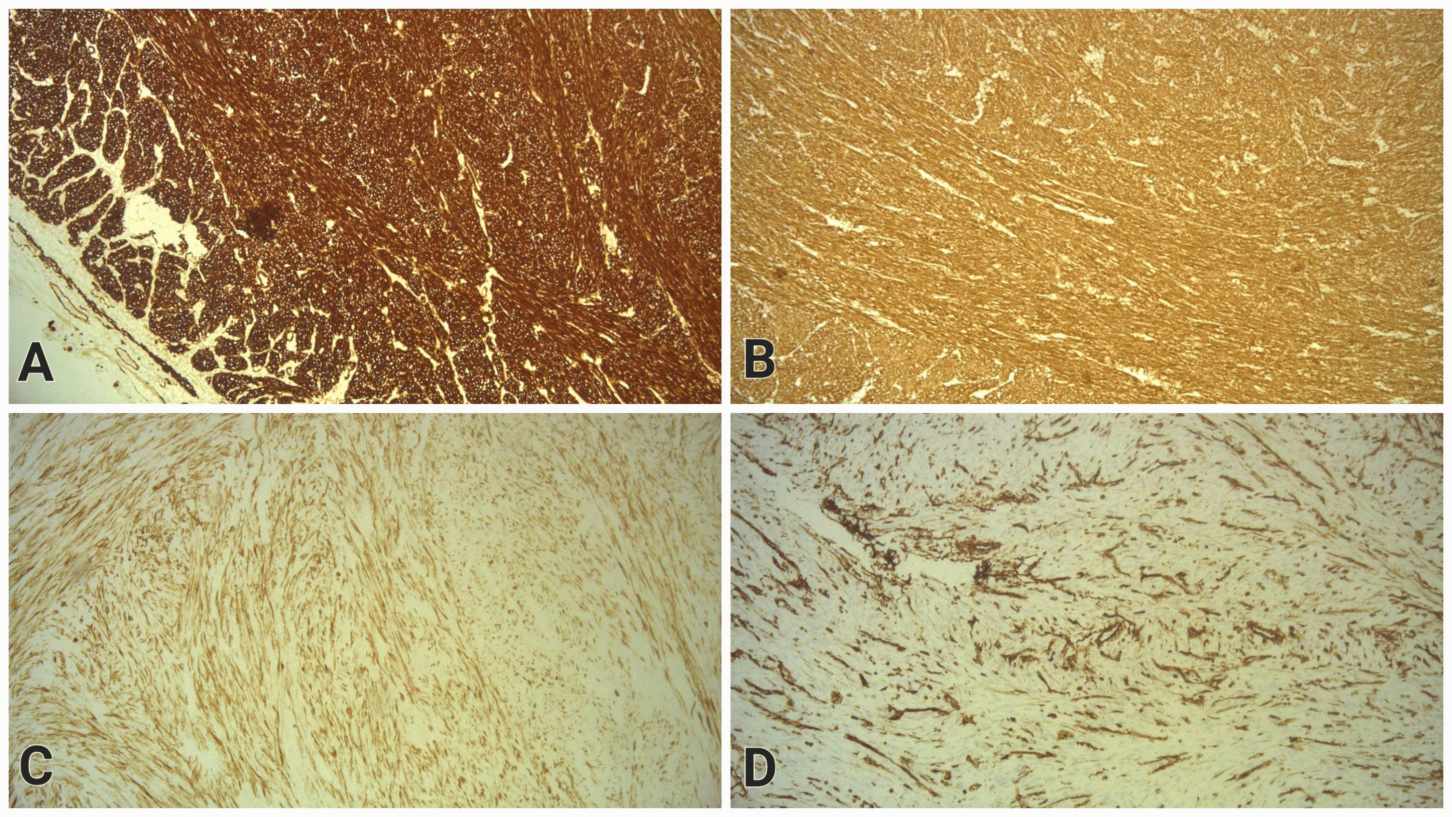
IHC displays diffusely positive reaction for h-caldesmon (A), SMA (B) and desmin (C) in the cells of the tumor parenchyma, while vimentin is expressed in some stromal cells (D), 100x IHC: Immunohistochemistry, SMA: smooth muscle actin

Immunohistochemistry (IHC) was performed to exclude different spindle cell pathologies, such as solitary fibrous tumor of the kidney, primary leiomyosarcoma, and sarcomatoid variants of renal cell carcinoma (RCC). IHC panel included cluster of differentiation-34 (CD-34), Ki-67, epithelial membrane antigen (EMA), soluble 100 protein (S100), pankeratin (CK AE1/AE3), smooth muscle actin (SMA), h-caldesmon, desmin, vimentin. Tumor cell cytoplasm was positive for h-caldesmon, SMA, and desmin (Figure 3A, 3B, 3C). Vimentin was expressed in some stromal cells (Figure 3D).

**Figure 4:**
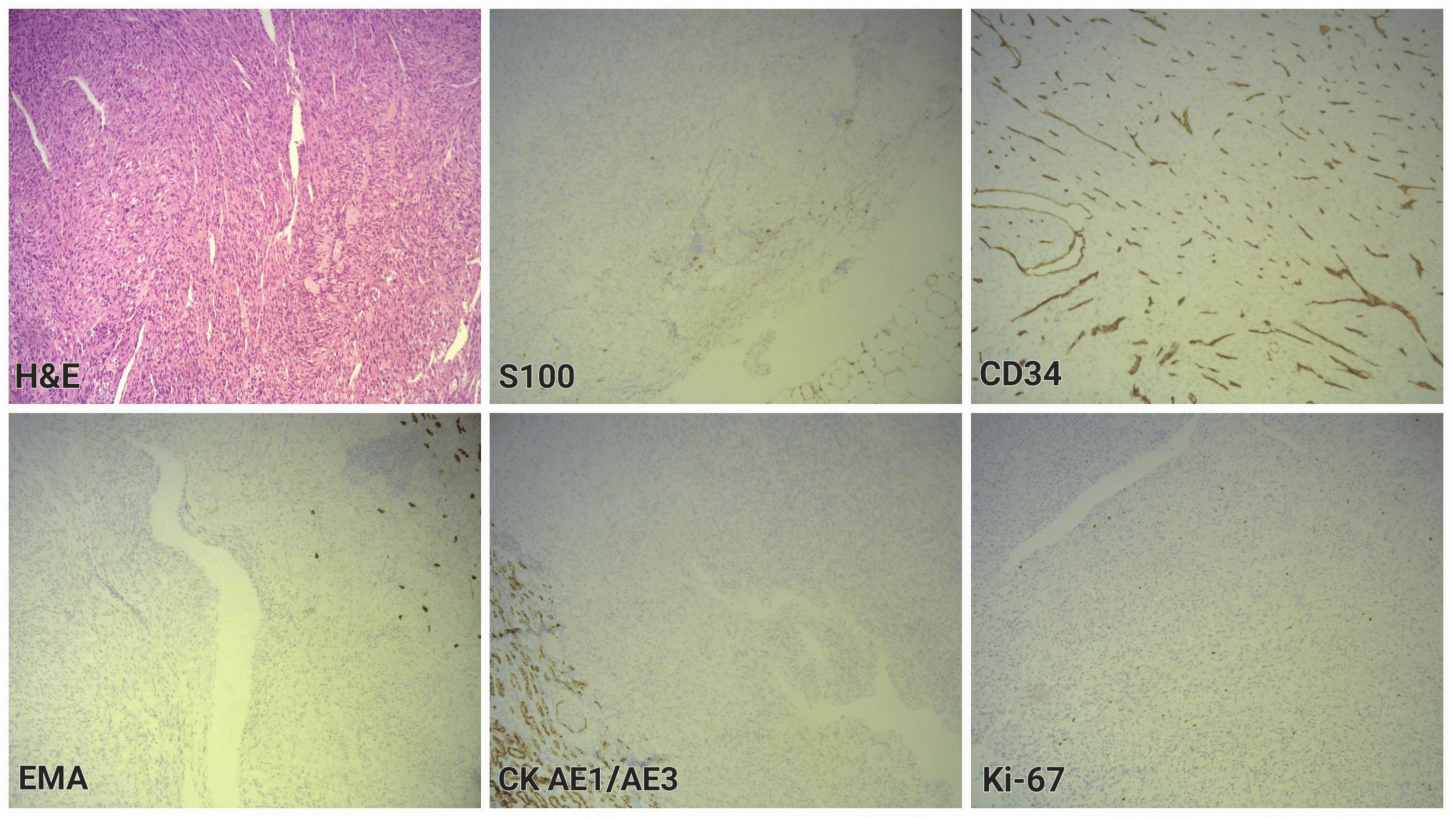
H&E staining of the specimen and IHC samples showing negative reaction in the tumor cells compared with internal controls, 100x S100 is expressed in perirenal adipose tissue. CD-34 expression is observed in vessel walls. EMA and CK AE1/AE3 are expressed in tubular epithelium. Ki-67 expression marks low mitotic activity within the tumor (<1%). H&E: Hematoxylin and eosin; IHC: Immunohistochemistry; S100: Soluble 100 protein; CD: Cluster of differentiation; EMA: Epithelial membrane antigen; CK AE1/AE3: Pankeratin; Ki-67: Kiel-67 (proliferation index)

Negative reaction by the tumor cells was reported for EMA, CK AE1/AE3, both expressed in the tubular epithelium. These negative epithelial markers excluded diagnoses with epithelial origin, such as sarcomatoid variant of RCC. S100, which was positive in the perirenal adipose tissue, showed no expression in the tumor cells. This finding excluded a neural crest origin of the tumor cells and neurofibroma or schwannoma as a potential diagnosis. CD-34 expression was noted only in vessel walls, which ruled out a solitary fibrous tumor. The proliferation index Ki-67 was evaluated as less than 1% (Figure 4). Considering the microscopic description and the IHC results, the neoplasm was interpreted as a renal leiomyoma.

## Discussion

In light of the rarity of renal leiomyomas, pathologists should consider differential diagnosis with precision. In challenging cases, immunohistochemical examination can be beneficial to distinguish between several spindle cell renal neoplasms, namely solitary fibrous tumor, leiomyosarcoma, sarcomatoid variant of RCC (Table 1). Other possible renal pathologies, included in the differential diagnosis, might be fat-poor angiomyolipoma, neurofibroma, schwannoma, renomedullary interstitial cell tumor, benign fibrous histiocytoma, primary angiosarcoma, mucinous tubular and spindle cell renal cell carcinoma (MTSRCC), although most of them have specific microscopic features which differ them morphologically from leiomyomas [4,5].

**Table 1:** Immunophenotype of leiomyomas compared with other spindle cell tumors [5-9] SMA: Smooth muscle actin; HMB: Human melanoma black; CD: Cluster of differentiation; Bcl-2: B-cell lymphoma 2; CK: Cytokeratins; STAT6: Signal transducer and activator of transcription 6; EMA: Epithelial membrane antigen; Ki-67: Kiel-67 (proliferation index); MSA: Muscle specific actin, RCC: Renal cell carcinoma; PAX8: Paired box gene 8; WT1: Wilms tumor 1 protein; S100: Soluble 100 protein (+): Positive; (+/–): Rarely positive; ↓: Low expression (<1%); ↑: High expression (>10%)

	Leiomyoma	Solitary fibrous tumor	Leiomyosarcoma	Sarcomatoid variant of RCC
Immunohistochemical profile	SMA (+)	CD-34 (+)	SMA (+)	CK (+)
	desmin (+)	Bcl-2 (+)	MSA (+)	RCC (+)
	h-caldesmon (+)	nuclear STAT6 (+)	desmin (+)	PAX8 (+)
	HMB-45 (+/–)	vimentin (+/–)	h-caldesmon (+)	CD-10 (+)
	Ki-67 ↓	CK (+/–)	vimentin (+)	EMA (+/–)
	-	SMA (+/–)	WT1 (+/–)	vimentin (+/–)
	-	desmin (+/–)	CK (+/–)	Ki-67 ↑
	-	S100 (+/–)	EMA (+/–)	-
	-	EMA (+/–)	CD-10 (+/–)	-
	-	Ki-67 ↓	Ki-67 ↑	-

Microscopically, leiomyomas exhibit benign characteristics such as no or minimal cellular atypia, no mitotic activity, and compressive behavior. In comparison, its malignant analogue demonstrates signs of malignant transformation. Abundant and aberrant mitotic figures, tumor necrosis, ability to infiltrate surrounding tissues, hemorrhages, and absence of granulation tissue around necrotic regions are indicative of leiomyosarcoma. Immunohistochemically, Ki-67 expression is usually low (<1%) in leiomyomas and other benign tumors and high (>10%) in malignancies [2].

Angiomyolipoma is a benign mesenchymal renal tumor composed of abnormally thick blood vessels, smooth muscle, and adipose tissue. Three types of angiomyolipoma are recognized: fat-rich (the most common), fat-poor, and epithelioid. They usually reveal IHC expression of SMA, MSA, human melanoma black-45 (HMB-45), Melan-A, S-100, and CD-117 [10].

Neurofibromas and schwannomas can be rarely observed in kidneys. These benign peripheral nerve sheath tumors strongly express S100 and are negative for smooth muscle molecular markers [11]. Common histological features of schwannomas are the Antoni A and Antoni B areas, which differ in cellularity and may contain Verocay bodies (parallel nuclear palisading separated by an anuclear zone) [12].

Renomedullary interstitial cell tumor, previously known as medullary fibroma, is also a benign neoplasm, mostly found incidentally because of its asymptomatic persistence. Immunohistochemically, the tumor has weak to moderate calponin and occasionally SMA expression [13].

Benign fibrous histiocytomas are divided into two types: superficial and deep. The former are located in the skin, and the latter, which are exclusively rare, involve internal organs (e.g., the kidneys). Their IHC profile includes diffuse vimentin and focal SMA expression in the tumor cells [14]. 

Primary renal angiosarcomas represent aggressive malignant neoplasms composed of pleomorphic spindle cells with a vasoformative growth pattern. Gourley et al. reported a case revealing positive erythroblast transformation-specific related gene (ERG) and CD-31 [4].

Since 2004, MTSRCC has been considered a distinct entity after being previously recognized as a subtype of RCC. Difficulties in the diagnostic process may appear in cases of variant patterns, such as in spindle cell predominant MTSRCC. According to data in the literature, positive staining for alpha-methylacyl-CoA racemase (AMACR), CK AE1/AE3, CK7, CK19, EMA, and vimentin is expected [15].

## Conclusions

The diagnosis of mesenchymal tumors of the kidneys represents a significant challenge for medical professionals. These neoplastic processes are less prevalent than epithelial tumors and encompass a diverse range of neoplasms, including benign and malignant variants. Some of these tumors are particularly rare in their specific locations. As illustrated in the case report, the differential diagnosis is inherently complex. Even in cases where tumors exhibit indolent clinical behaviors or evident histopathology, specialized studies are often necessary to exclude rare variants of malignant mesenchymal tumors. The description of rare nosological entities enhances the body of knowledge and facilitates the processes of diagnosis.
